# Association between Alzheimer’s disease genes and trajectories of cognitive function decline in Han Chinese in Taiwan

**DOI:** 10.18632/aging.203204

**Published:** 2021-07-02

**Authors:** Tsung-Jen Hsieh, Wei-Ju Lee, Yi-Chu Liao, Chih-Cheng Hsu, Yao-Hwei Fang, Tzu-Yu Chen, Yung-Shuan Lin, I-Shou Chang, Shuu-Jiun Wang, Chao A. Hsiung, Jong-Ling Fuh, Alzheimer’s Disease Neuroimaging Initiative

**Affiliations:** 1 Institute of Population Health Sciences, National Health Research Institutes, Miaoli, Taiwan; 2 School of Medicine, I-Shou University, Kaohsiung, Taiwan; 3 Neurological Institute, Taichung Veterans General Hospital, Taichung, Taiwan; 4 Faculty of Medicine, National Yang-Ming University Schools of Medicine, Taipei, Taiwan; 5 Dementia Center, Taichung Veterans General Hospital, Taichung, Taiwan; 6 Center for Geriatrics and Gerontology, Taichung Veterans General Hospital, Taichung, Taiwan; 7 Brain Research Center, National Yang-Ming University, Taipei, Taiwan; 8 Department of Neurology, Neurological Institute, Taipei Veterans General Hospital, Taipei, Taiwan; 9 National Institute of Cancer Research, National Health Research Institutes, Miaoli, Taiwan

**Keywords:** Alzheimer's disease, trajectory analysis, APOE ɛ4 allele, ABCA7 gene, SORL1 gene

## Abstract

Genetic background has been considered one of the important contributors to the rate of cognitive decline among patients with Alzheimer’s disease (AD). We conducted a 4-year longitudinal follow-up study, recruited 255 AD and 44 mild cognitive impairment (MCI) patients, and used a data-driven trajectory analysis to examine the influence of selected AD risk genes on the age for and the rate of cognitive decline in Han Chinese population. Genotyping of selected single-nucleotide polymorphisms in the *APOE*, *ABCA7*, *SORL1*, *BIN1*, *GAB2*, and *CD33* genes was conducted, and a Bayesian hierarchical model was fitted to analyze the trajectories of cognitive decline among different genotypes. After adjusting for sex and education years, the *APOE* ε4 allele was associated with an earlier mean change of −2.39 years in the age at midpoint of cognitive decline, the G allele in *ABCA7* rs3764650 was associated with an earlier mean change of −1.75 years, and the T allele in *SORL1* rs3737529 was associated with a later mean change of 2.6 years. Additionally, the rate of cognitive decline was associated with the *APOE* ε4 allele and *SORL1* rs3737529. In summary, *APOE* and *SORL1* might be the most important genetic factors related to cognitive decline in Han Chinese population.

## INTRODUCTION

Alzheimer’s disease (AD) is a neurodegenerative disease characterized by progressive cognitive decline and functional impairment. The rate of cognitive decline varies substantially among different AD patients. There are many factors that contribute to the rate of cognitive decline, such as age of disease onset, gender, education, extrapyramidal signs, behavioral disorders, vascular risk factors and the most known AD susceptibility genetic locus, *apolipoprotein E* (*APOE*) genotypes [1, 2]. Among these factors, genetic background is one of the most important contributors to the rate of cognitive decline. The results from previous studies have shown that having at least one *APOE* ε4 allele was associated with faster cognitive decline in cognitively healthy older Taiwanese adults [3], and *PDE7A* and *MTFR1* genes were associated with the rate of age-related cognitive decline [4]. Moreover, *BDNF* Val66Met was associated with faster cognitive decline and greater hippocampal atrophy in preclinical AD patients [5]. However, some evidence has revealed that these risk genes may act differently in different populations. For example, in a meta-analysis, AD was significantly associated with variants in *ABCA7* in African American participants and with other genes that have been associated with AD in individuals of European ancestry [6]. In addition, the *ABCA7* rs3764650 GG genotype was reported to increase the risk of AD in Caucasians and African Americans, but a protective effect was found in the Han Chinese population in our prior study [7]. In a study validating genome-wide association studies (GWAS)-identified risk loci based on Caucasians populations showed that not all of these risk loci were linked to the risk of AD in Han Chinese populations [8]. Different results regarding *APOE* was also found among white, black or Hispanic respondents [9]. If the association between genotypes and AD risk varies by race/ethnicity, then this may be the case for the rate of cognitive decline as well. Hence, the effect of genetic factors on the rate of cognitive decline should be addressed in different ethnic groups [10].

Recent GWAS have identified more than 20 AD susceptibility loci, including *CR1*, *CLU*, *PICALM*, *BIN1*, *CD2AP*, *CD33*, *EPHA1*, *MS4A6A*/*MS4A4E*, *SORL1*, *GAB2*, *ABCA7*, etc [11–18]. However, most of these studies have conducted in Caucasian population and data about the genetics of AD from other populations has been relatively limited [10]. In Han Chinese or Asian population, common variants in *GCH1*, *KCNJ15* [19], and rare missense variant in the *C7* genes [20] were also identified by whole-genome sequencing and whole-exome sequencing studies. In our previous studies, we identified *ABCA7* rs3764650 and *SORL1* rs1784933 as being associated with the risk of AD in Han Chinese individuals in Taiwan [7, 21], the association between *BIN1* rs744373 and AD was reported in Asian populations [8], but the effect of these AD risk genes on the rate of cognitive decline is not clear. In this study, we used a data-driven trajectory analysis to examine the influence of selected AD risk genes on the age for and the rate of cognitive decline in the Han Chinese population.

## RESULTS

A total of 299 patients—255 with AD and 44 with MCI—were included in this study. The baseline demographic and genetic data of the study participants are shown in Table 1. At baseline, the mean age of the study participants at study entry was 78.4 ± 7.0 years, 51.8% of the participants were women, and the mean MMSE score was 20.0 ± 4.9. The genotypic distributions of all SNPs were consistent with Hardy-Weinberg equilibrium (all p-values > 0.05).

**Table 1:** Baseline demographics and genetic characteristics of the study participants.

**Variables**	**Total patients (*n* = 299)**	**AD patients (*n* = 255)**	**MCI patients (*n* = 44)**
Age (years)	78.4 ± 7.0	79.3 ± 6.5	73.3 ± 7.7
Sex (women)	155 (51.8)	134 (52.6)	21 (47.7)
Education (years)	9.5 ± 4.6	9.2 ± 4.6	11.5 ± 4.3
MMSE (scores)	20.0 ± 4.9	19.2 ± 4.7	24.7 ± 3.0
*APOE* genotypes			
ε2ε2/ε2ε3/ε3ε3	195 (65.2)	166 (65.1)	29 (65.9)
ε2ε4/ε3ε4	93 (31.1)	81 (31.8)	12 (27.3)
ε4ε4	11 (3.7)	8 (3.1)	3 (6.8)
*ABCA7* rs3764650			
TT	135 (45.2)	111 (43.5)	24 (54.6)
TG	128 (42.8)	113 (44.3)	15 (34.1)
GG	36 (12.0)	31 (12.2)	5 (11.4)
*SORL1* rs3737529			
CC	183 (61.2)	150 (58.8)	33 (75.0)
CT	99 (33.1)	90 (35.3)	9 (20.5)
TT	17 (5.7)	15 (5.9)	2 (4.6)
*SORL1* rs1784933			
AA	139 (46.5)	114 (44.7)	25 (56.8)
AG	134 (44.8)	118 (46.3)	16 (36.4)
GG	26 (8.7)	23 (9.0)	3 (6.8)
*SORL1* rs2298813			
GG	251 (84.0)	215 (84.3)	36 (81.8)
GA	45 (15.1)	38 (14.9)	7 (15.9)
AA	3 (1.0)	2 (0.8)	1 (2.3)
*BIN1* rs744373			
AA	104 (34.8)	90 (35.3)	14 (31.8)
AG	142 (47.5)	117 (45.9)	25 (56.8)
GG	53 (17.7)	48 (18.8)	5 (11.4)
*GAB2* rs2373115			
CC	139 (46.5)	125 (49.0)	14 (31.8)
CA	120 (40.1)	97 (38.0)	23 (52.3)
AA	40 (13.4)	33 (12.9)	7 (15.9)
*CD33* rs3865444			
CC	203 (67.9)	173 (67.8)	30 (68.2)
CA	82 (27.4)	71 (27.8)	11 (25.0)
AA	14 (4.7)	11 (4.3)	3 (6.8)

The data are expressed as the mean ± SD or n (%).

Abbreviations: AD, Alzheimer’s disease; MCI, mild cognitive impairment; MMSE, Mini-Mental State Examination; *APOE*, apolipoprotein E.

After adjusting for sex and education years, the effects of all AD genetic markers on the parameters *M* and *R* of the trajectories of cognitive function decline based on the MMSE are shown in Table 2. Regarding the age at the midpoint of the cognitive function decline, one copy of the ε4 allele in the *APOE* gene was significantly associated with an earlier mean change of −2.39 years (95% CI: −4.12, −0.64); one copy of the G allele in *ABCA7* rs3764650 was significantly associated with an earlier mean change of −1.75 years (95% CI: −3.09, −0.37); one copy of the T allele in *SORL1* rs3737529 was significantly associated with a later mean change of 2.60 years (95% CI: 0.38, 4.88). Moreover, the rate of cognitive decline was associated with the *APOE* gene (posterior mean = 0.96, 95% CI: 0.39, 1.57) and *SORL1* rs3737529 (posterior mean = 0.58, 95% CI: 0.05, 1.14). No statistically significant associations were observed between either the age at midpoint or the rate of cognitive decline and the genetic markers of *SORL1* rs1784933, *SORL1* rs2298813, *BIN1* rs744373, *GAB2* rs2373115, and *CD33* rs3865444.

**Table 2:** Effects of genetic markers on the age at the midpoint of cognitive function decline (*M*) and the rate of cognitive function decline (*R*) parameters based on the MMSE after adjusting for sex and education years (*n* = 299).

**Covariates**	**Minor allele**	**Regression coefficients on *M***			**Regression coefficients on *R***	
		**Posterior mean**	**95% CI**		**Posterior mean**	**95% CI**
Sex (female vs. male)	—	−1.11	(−3.05, 0.85)		0.62	(0.16, 1.14)*
Education years	—	0.37	(0.15, 0.58)*		−0.05	(−0.09, 0.001)
*APOE*	ε4	−2.39	(−4.12, −0.64)*		0.96	(0.39, 1.57)*
*ABCA7* rs3764650	G	−1.75	(−3.09, −0.37)*		−0.20	(−0.55, 0.14)
*SORL1* rs3737529	T	2.60	(0.38, 4.88)*		0.58	(0.05, 1.14)*
*SORL1* rs1784933	G	1.04	(−1.02, 3.03)		0.12	(−0.38, 0.58)
*SORL1* rs2298813	A	0.91	(−1.58, 3.52)		−0.06	(−0.62, 0.55)
*BIN1* rs744373	G	−0.46	(−1.74, 0.81)		−0.20	(−0.47, 0.09)
*GAB2* rs2373115	A	−0.25	(−1.58, 1.07)		0.10	(−0.25, 0.47)
*CD33* rs3865444	A	1.43	(−0.20, 3.08)		−0.07	(−0.49, 0.39)

Note: The full sample of 255 AD and 44 MCI patients at baseline were analyzed.

Abbreviations: MMSE, Mini-Mental State Examination; CI, credible interval.

*Statistically significant with 95% credible interval.

To assess which variable provided the best fit to the longitudinal data from the MMSE, we then implemented a Bayesian variable selection using the Gibbs variable selection method. The results of model selection among the eight genetic markers are summarized in Table 3. Sex and education variables were included in each model. The results revealed that, compared with other models, the *APOE* gene and *SORL1* rs3737529 in model 1 had the best fit to the data based on posterior model probabilities. We again evaluated these two genetic markers in the Bayesian hierarchical model. Significant associations and estimated directions were consistent with the full model that included all genetic markers (Table 4). Moreover, to check the fitness of a Bayesian model, we then calculated a posterior predictive p-value. The value was 0.937, which indicated that the fitted model was adequate to describe longitudinal data from the MMSE.

**Table 3:** Model selection among the eight genetic markers after adjusting for sex and education years (*n* = 299).

**Models**	**Variables in the model**	**Posterior model probabilities**
Model 1	*APOE*, *SORL1* rs3737529	0.8956
Model 2	*APOE*, *SORL1* rs3737529, *SORL1* rs1784933	0.0548
Model 3	*APOE*, *SORL1* rs3737529, *CD33* rs3865444	0.0243
Model 4	*APOE*, *SORL1* rs3737529, *BIN1* rs744373	0.0113
Model 5	*APOE*, *SORL1* rs3737529, *GAB2* rs2373115	0.0062
Model 6	*APOE*, *SORL1* rs3737529, *SORL1* rs2298813	0.0056
Model 7	*APOE*, *SORL1* rs3737529, *SORL1* rs1784933, *CD33* rs3865444	0.0023
Model 8	*APOE*, *SORL1* rs3737529, *SORL1* rs1784933, *SORL1* rs2298813	0.0001

Note: The full sample of 255 AD and 44 MCI patients at baseline were analyzed.

**Table 4:** Effects of *APOE* and *SORL1* rs3737529 on the age at the midpoint of cognitive function decline (*M*) and the rate of cognitive function decline (*R*) parameters based on the MMSE after Bayesian variable selection (*n* = 299).

**Covariates**	**Minor allele**	**Regression coefficients on *M***			**Regression coefficients on *R***	
		**Posterior mean**	**95% CI**		**Posterior mean**	**95% CI**
Sex (female vs. male)	—	−1.05	(−3.00, 0.94)		0.61	(0.12, 1.12)*
Education years	—	0.38	(0.18, 0.59)*		−0.04	(−0.10, 0.01)
*APOE*	ε4	−2.79	(−4.46, −1.09)*		0.93	(0.44, 1.49)*
*SORL1 rs3737529*	T	1.53	(0.02, 3.04)*		0.41	(0.02, 0.87)*

Note: The full sample of 255 AD and 44 MCI patients at baseline were analyzed.

Abbreviations: MMSE, Mini-Mental State Examination; CI, credible interval.

*Statistically significant with 95% credible interval.

**Figure 1:**
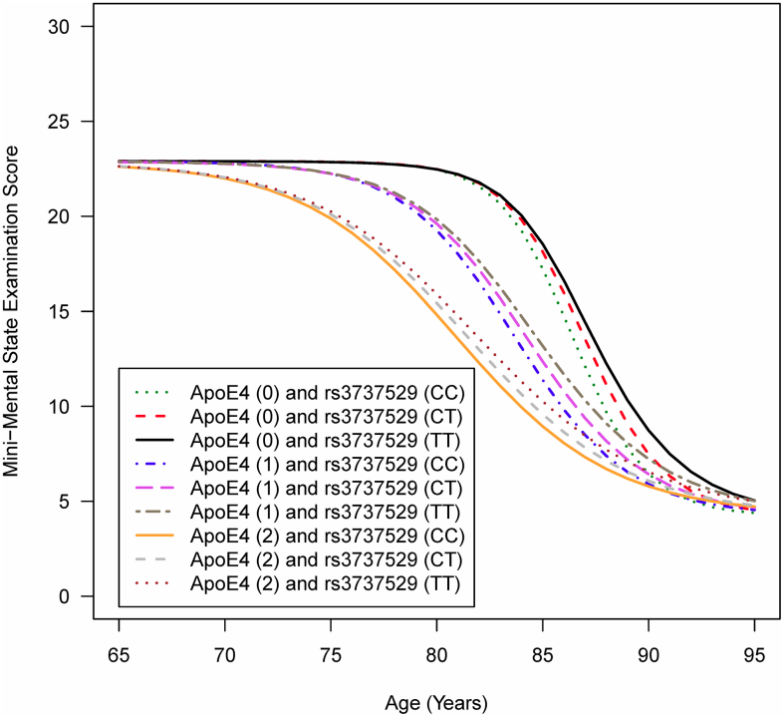
**Mean trajectories of cognitive function for individuals carrying specific genotypes in the APOE gene and SORL1 rs3737529 SNPa.** AIn the figure legend, ApoE4 (0/1/2) means that individuals are carrying zero, one, or two copies of the ε4 allele in the APOE gene; rs3737529 (CC/CT/TT) means that individuals are carrying the CC, CT, or TT genotypes in SORL1 rs3737529. Note: The full sample of 255 AD and 44 MCI patients at baseline were analyzed.

Figure 1 depicts the mean trajectories of cognitive function based on the MMSE for individuals of the same sex and education level carrying specific genotypes in the *APOE* gene and *SORL1* rs3737529. Figure 1 shows that a more rapid decline in cognitive function was observed for individuals possessing two copies of the ε4 allele in the *APOE* gene and the CC genotype in *SORL1* rs3737529. In other words, people with the ε4 allele and C allele tended to have earlier cognitive function decline than those with the non-ε4 allele and the T allele.

In order to test robustness of our results, we excluded 16 stable MCI patients, who had not progressed to AD during the observation period, from the full sample, and used the subset of 283 samples to validate the results obtained from the full samples. After adjusting for sex and education years, similar results showed that the *APOE* ε4 allele was significantly associated with an earlier mean change of −2.10 years in the age at midpoint of cognitive decline, the G allele in *ABCA7* rs3764650 was significantly associated with an earlier mean change of −1.74 years, and the T allele in *SORL1* rs3737529 was significantly associated with a later mean change of 2.65 years. Moreover, the rate of cognitive decline was associated with the *APOE* ε4 allele and *SORL1* rs3737529 (Supplementary Table 1). Model selection and estimation of effects using 283 patients are summarized in Supplementary Tables 2, 3. No matter whether the stable MCI patients were included in the analyzed sample or not, the results shown that *APOE* gene and *SORL1* rs3737529 were associated with the age at midpoint of cognitive function decline and the rate of cognitive function decline.

To validate our results with an independent ADNI cohort, we also fitted a Bayesian hierarchical model to depict the trajectories of cognitive function decline using the ADNI data. The effects of all genetic markers on the parameters *M* and *R* of the trajectories of cognitive function decline based on the MMSE after adjusting for sex and education years are shown in Supplementary Table 4. The results revealed that only the *APOE* gene was associated with the age at midpoint of cognitive function decline.

## DISCUSSION

In this study, we demonstrated that the *APOE*, *ABCA7*, and *SORL1* genes were associated with cognitive function decline in the Han Chinese population. The results revealed that one copy of the ε4 allele in the *APOE* gene and one copy of the G allele in *ABCA7* rs3764650 were significantly associated with earlier midpoints of cognitive decline; one copy of the T allele in *SORL1* rs3737529 was significantly associated with a later midpoint of cognitive decline. Additionally, the *APOE* gene and *SORL1* rs3737529 were associated with the rate of cognitive decline. The T allele in *SORL1* rs3737529 seems to be protective against cognitive decline independent of *APOE* genotype. However, in ADNI population, only *APOE* gene was associated with the age at midpoint of cognitive function decline.

One prior study in North America found that *CLU* and *CR1* were associated with more rapid cognitive decline and that *PICALM* was associated with an earlier age at the midpoint of cognitive decline in patients with AD. These associations remained after accounting for the effects of *APOE* and demographic factors [22]. The present study provides additional evidence that different populations have their own risk genes that may influence cognitive decline in AD and MCI patients and demonstrates that measuring cognitive trajectories is a way to test the genetic associations with both age of and rate of cognitive decline.

The relationship between *APOE* ε4 carriage and the rate of cognitive decline in AD patients has been examined in several studies. However, their results have been inconsistent [23], which might have been due to the distinct genetic effects in different populations and complex interactions between the *APOE* gene and other genetic and vascular risk factors [2]. Previous studies have shown that the effect of the *APOE* gene on the risk of cognitive decline and dementia was modified by other genetic factors, including *ABCA7* [7], *SORL1* [21, 24], *PICALM* [25], *CR1* [26], *BIN1* [16], and *TREM2* [27], showing complex gene-gene interactions [28]. Although the rate of cognitive decline and the risk of AD are not identical, the presence of complex gene-gene interactions of the *APOE* gene in both situations should be considered. A previous study using data from the Taiwan Longitudinal Study of Aging showed an association between the *APOE* genotype and the rate of cognitive decline in a predominantly Han Chinese population [3]. In the present study, the *APOE* gene ε4 allele was significantly associated with an earlier midpoint of cognitive decline in AD and MCI patients in both the Han Chinese population in Taiwan and ADNI population in America and associated with a rapid rate of cognitive decline in the Han Chinese population.

*ABCA7* is an ATP-binding cassette transporter protein mainly expressed in microglia and neurons [29, 30]. The functions of *ABCA7* include lipid metabolism, regulation of phagocytosis, and amyloid β (Aβ) production and clearance [31]. The minor allele of rs3764650 in *ABCA7* was associated with increased neuritic plaque formation [32] and decreased Aβ levels in cerebrospinal fluid [33]. Clinically, the *ABCA7* rs3764650 minor allele was associated with cortical and hippocampal atrophy [34] and with a later age at onset and shorter disease duration [35]. In accordance with these findings in previous studies, our study demonstrated that the minor allele of rs3764650 in *ABCA7* was associated with an earlier midpoint of cognitive function decline, which suggested that the *ABCA7* gene affects the clinical course of AD from the preclinical stage to the dementia stage.

*SORL1* encodes a multidomain-containing, membrane-bound receptor involved in endosomal sorting of proteins between the trans-Golgi network, endosomes and the plasma membrane [36]. In AD, the *SORL1* encoded receptor interacts with the amyloid precursor protein (APP) and the Aβ peptide [37], participating in APP trafficking and processing and Aβ destruction [38]. Previous studies have also shown that two *SORL1* polymorphisms (rs3824968-A allele and rs2282649-T allele) were related to decreased cerebrospinal fluid (CSF) concentrations of Aβ42 and Aβ40 [39], the rs2070045-G allele was associated with increased CSF tau and more hippocampal atrophy [40], and *SORL1* rs11218343 was associated with cognitive performance [41]. In our previous study, *SORL1* rs1784933 and rs2298813 were associated with AD and MCI risk in the Han Chinese population in Taiwan [21], and in the present study, *SORL1* rs3737529 was associated with the midpoint and the rate of cognitive decline in AD and MCI patients in Han Chinese population. These results suggest that *SORL1* plays many different roles in AD pathogenesis that are significantly related to clinical manifestations. The associations between *ABCA7* as well as *SORL1* and cognitive function decline were found in Han Chinese population, but not in the ADNI data. It might also suggest that these risk genes may act differently in different populations [6–9].

There are some limitations in this study. First, no other cohort can validate our findings in Taiwan currently. Other longitudinal follow-up studies need to be conducted to test these results. Second, the diagnoses of MCI and AD were made according to the clinical criteria without biomarker evidence of Aβ and tau, which may have influenced the diagnostic accuracy. Third, we used the MMSE to evaluate longitudinal cognitive changes, which might underestimate cognitive decline. Forth, we only tested a limited number of SNPs based on the evidence of significant genetic association in some previous studies. Other SNPs may also be associated with the trajectory of cognitive decline, and the genetic effects might be different in different populations. Finally, the sample size of our cohort is relatively small compared to the other longitudinal cohort (such as ADNI cohort). This may limit the generalizability of our findings to general population.

In summary, we used a Bayesian approach to examine the genetic effects on the trajectory of cognitive decline in AD and MCI patients in the Han Chinese population. The carriage of the *APOE* ε4 genotype and the G allele in *ABCA7* rs3764650 were significantly associated with an earlier midpoint of cognitive decline. In contrast, the T allele in *SORL1* rs3737529 was significantly associated with a later midpoint of cognitive decline. Additionally, the *APOE* gene and *SORL1* rs3737529 were associated with the rate of cognitive function decline.

## MATERIALS AND METHODS

### Subjects

Participants were recruited from two teaching hospitals in the Biosignature Study of Alzheimer’s Disease (BSAD). The BSAD is carried out as a subproject of Taiwan Biobank [42] and has been designed as a prospective longitudinal follow-up study at 1-year intervals since 2012 to identify potential biomarkers for early diagnosis of AD in the Han Chinese population. The collected data included patients’ neuropsychological test outcomes, blood biomarkers, brain magnetic resonance imaging, and related clinical characteristics. Overall, 255 AD patients and 44 mild cognitive impairment (MCI) patients with at least four measurements were selected for modeling trajectories of cognitive function decline. For the 44 MCI patients at baseline, 28 patients (63.6%) would progress to AD during the consecutive follow-up period. An AD diagnosis was made during a multidisciplinary consensus meeting according to the clinical criteria for probable AD described by the National Institute on Aging–Alzheimer’s Association [43]. A diagnosis of MCI was made according to the revised criteria established from the consensus report [44, 45]. The cutoff value for the diagnosis of MCI was set at 1.5 standard deviations below the age-adjusted norm for the logical memory test of the Wechsler Memory Scale III [46]. Other inclusion criteria included an age at onset greater than 60 years and the availability of a caregiver who could provide collateral patient history. The exclusion criteria were significant neurological diseases other than AD that may affect cognition, including Parkinson’s disease, vascular cognitive impairment, normal pressure hydrocephalus, brain tumor, progressive supranuclear palsy, seizure disorder, subdural hematoma, and multiple sclerosis, a history of significant head trauma followed by persistent neurologic deficits, or other known significant structural brain abnormalities. All patients received a standardized evaluation that included a clinical interview, neuropsychological assessment, laboratory tests and brain magnetic resonance imaging. The institutional review boards of each participating hospital approved the protocol and informed consent form for this study. All participants or their legal representatives signed informed consent forms at study participation.

### Cognitive test

Global cognition was annually assessed using the Mini-Mental State Examination (MMSE) [47], which was carried out to depict the trajectory of cognitive function for each participant. The total scores ranged between 0 and 30, and lower scores revealed poorer cognitive performance. The Clinical Dementia Rating (CDR) [48] was administered to determine the severity of dementia. Additionally, the 12-item memory test, modified 15-item Boston Naming Test, category verbal fluency test, and forward and backward digit span test were used at study entry to assess short-term memory, language, executive function, attention and working memory, respectively. Longitudinal follow-up was performed in AD and MCI patients at one-year intervals with the MMSE and the CDR assessment.

### Genotypic data

For each participant, a Gentra Puregene kit (Qiagen, Hilden, Germany) was utilized to extract genomic DNA from whole blood samples based on standard protocols. Previous studies have reported that AD is associated with several genes, including *APOE*, *ABCA7* [7, 11, 49], *SORL1* [17, 21, 50, 51], *BIN1* [52], *GAB2* [18], and *CD33* [11, 53]. Two single-nucleotide polymorphisms (SNPs), rs429358 and rs7412, were selected to genotype the ε2, ε3, and ε4 alleles of the *APOE* gene [54]. Seven SNPs were selected and considered to serve as genetic markers for these candidate genes on the basis of (1) our previous study showing *ABCA7* rs3764650, *SORL1* rs1784933, and *SORL1* rs2298813 associated with the risk of AD or MCI in Han Chinese individuals in Taiwan [7, 21] (2) *SORL1* rs3737529 being one of the most significant SNPs in Asian population [17] (3) *BIN1* rs744373 and *CD33* rs3865444 being the AlzGene Top Results (alzgene.org) and being associated with the risk of AD in Caucasian and Han Chinese population [8, 11] (4) *GAB2* 2373115 being associated with the AD risk with an odds ratio of 4.06 [18].

Genotyping of all SNPs was accomplished using the TaqMan genotyping assay (Applied Biosystems, Foster City, CA, USA) (Supplementary Table 5). Polymerase chain reaction (PCR) was carried out using 96-well microplates with an ABI 7500 real-time PCR system (Applied Biosystems). Allele discrimination was performed by identifying fluorescence using SDS software version 1.2.3 (Applied Biosystems).

### ADNI dataset

ADNI data used in this study were obtained from the Alzheimer’s Disease Neuroimaging Initiative (ADNI) database (adni.loni.usc.edu) [55, 56]. The ADNI was launched in 2003 as a public-private partnership, led by Principal Investigator Michael W. Weiner, MD. The primary goal of ADNI has been to test whether serial magnetic resonance imaging (MRI), positron emission tomography (PET), other biological markers, and clinical and neuropsychological assessment can be combined to measure the progression of MCI and early AD. For up-to-date information, see http://www.adni-info.org.

To compare with ADNI cohort, a total of 385 ADNI patients in the ADNI-1, ADNI-GO, or ADNI-2 phases—150 with AD and 235 with MCI and then progression to AD—with at least four MMSE measurements and genotypic data were selected to analyze the trajectories of cognitive function decline. Because the three candidate genetic markers—rs3737529 and rs1784933 in *SORL1*, and rs2373115 in *GAB2*— were not genotyped in the ADNI-1 and/or ADNI-GO/2 data, we imputed the two datasets separately to the 1000 Genomes Project (1000 Genomes Phase 3 v5 used as the reference panel, SHAPEIT as the phasing, and EUR as the population) using Minimac3 on the Michigan Imputation Server [PMID: 27571263].

### Statistical analysis

For each SNP, Hardy-Weinberg equilibrium was checked using the goodness-of-fit test in AD and MCI subjects. A Bayesian hierarchical model with a four-parameter logistic curve [22] was fitted to depict the trajectories of cognitive function decline for patients with AD or MCI at baseline (*n* = 299). This model included four major parameters: the asymptotic value of cognitive function at lower ages (*A*), the asymptotic value of cognitive function at higher ages (*B*), the age at the midpoint of cognitive function decline (*M*) between *A* and *B*, and the rate of cognitive function decline (*R*) from *A* to *B*. In the model, random effects on parameters *A* and *B* were assumed to model the variation between subjects. In addition, to consider the effects of genetic markers, linear combinations of these covariates on parameters *M* and *R* were modeled adjusting for sex and education years, and an additive mode of inheritance was assumed to code the number of copies of the minor allele for each genetic marker. The distributions of observed measurements and the prior distributions of model parameters followed the assumption proposed by Sweet et al. [22]. Bayesian inference was based on an initial 2,000 iteration burn-in and a 20,000 iteration run using Gibbs sampling.

To identify an adequate fitting model of genetic markers on trajectories of cognitive function decline, model selection was carried out using Gibbs variable selection [57]. The statistical significance of all tests was evaluated using a 95% credible interval. The adequacy of the fitted Bayesian model to the data was checked by posterior predictive p-value [58]. All Bayesian analyses were performed using the open-source software WinBUGS version 1.4.3 [59]. Besides, not all MCI patients will develop clinically defined AD during the follow-up period. Therefore, we excluded 16 stable MCI patients without progression to AD and used the subset of 283 samples to validate the results obtained from the full samples. In addition, a total of 385 ADNI patients with AD or with MCI and then progression to AD were fitted to validate the trajectories of cognitive function decline obtained from our BSAD patients.

## Supplementary Material

Supplementary Tables
