# Supplementary material for: Association between Alzheimer’s disease genes and trajectories of cognitive function decline in Han Chinese in Taiwan
Source: Aging (Albany NY). 2021 Jul 2;13(13):17237–52. doi: 10.18632/aging.203204 (PMC8312434; doi:10.18632/aging.203204)
Supplement: Supplementary Tables [file aging-13-203204-s001.pdf]

SUPPLEMENTARY TABLES

Supplementary Table 1. Effects of genetic markers on the age at the midpoint of cognitive function decline (*M*) and the rate of cognitive function decline (*R*) parameters based on the MMSE after adjusting for sex and education years (*n* = 283).

Covariates	Minor allele	Regression coefficients on *M*		Regression coefficients on *R*	
		Posterior mean	95% CI	Posterior mean	95% CI
Sex (female vs. male)	—	−1.02	(−2.93, 0.92)	0.64	(0.23, 1.11)*
Education years	—	0.39	(0.18, 0.61)*	−0.04	(−0.09, −0.003)*
APOE	ε4	−2.10	(−3.79, −0.34)*	0.90	(0.37, 1.42)*
ABCA7 rs3764650	G	−1.74	(−3.16, −0.29)*	−0.20	(−0.51, 0.13)
SORL1 rs3737529	T	2.65	(0.41, 4.90)*	0.56	(0.01, 1.07)*
SORL1 rs1784933	G	1.16	(−0.91, 3.22)	0.12	(−0.33, 0.52)
SORL1 rs2298813	A	0.88	(−1.78, 3.61)	−0.005	(−0.60, 0.71)
BIN1 rs744373	G	−0.47	(−1.79, 0.86)	−0.16	(−0.43, 0.11)
GAB2 rs2373115	A	−0.35	(−1.69, 0.99)	0.09	(−0.23, 0.40)
CD33 rs3865444	A	1.32	(−0.34, 2.95)	−0.05	(−0.41, 0.36)

Note: The subset of 255 AD and 28 MCI progression to AD patients were analyzed.
Abbreviations: MMSE, Mini-Mental State Examination; CI, credible interval.
*Statistically significant with 95% credible interval.

Supplementary Table 2. Model selection among the eight genetic markers after adjusting for sex and education years (*n* = 283).

Models	Variables in the model	Posterior model probabilities
Model 1	*APOE*, *SORL1* rs3737529	0.8988
Model 2	*APOE*, *ABCA7* rs3764650, *SORL1* rs3737529, *SORL1* rs1784933	0.0565
Model 3	*APOE*, *SORL1* rs3737529, *CD33* rs3865444	0.0216
Model 4	*APOE*, *SORL1* rs3737529, *SORL1* rs1784933	0.0131
Model 5	*APOE*, *SORL1* rs3737529, *SORL1* rs2298813	0.0066
Model 6	*APOE*, *SORL1* rs3737529, *GAB2* rs2373115	0.0024
Model 7	*APOE*, *SORL1* rs3737529, *BIN1* rs744373, *GAB2* rs2373115	0.0009
Model 8	*APOE*, *SORL1* rs3737529, *BIN1* rs744373	0.0002

Note: The subset of 255 AD and 28 MCI progression to AD patients were analyzed.

Supplementary Table 3. Effects of *APOE* and *SORL1* rs3737529 on the age at the midpoint of cognitive function decline (*M*) and the rate of cognitive function decline (*R*) parameters based on the MMSE after Bayesian variable selection (*n* = 283).

Covariates	Minor allele	Regression coefficients on *M*		Regression coefficients on *R*	
		Posterior mean	95% CI	Posterior mean	95% CI
Sex (female vs. male)	—	−0.89	(−2.88, 1.12)	0.59	(0.12, 1.12)*
Education years	—	0.40	(0.19, 0.62)*	−0.04	(−0.09, 0.003)
APOE	ε4	−2.50	(−4.25, −0.73)*	0.82	(0.37, 1.30)*
SORL1 rs3737529	T	1.58	(0.01, 3.23)*	0.43	(0.06, 0.80)*

Note: The subset of 255 AD and 28 MCI progression to AD patients were analyzed.
Abbreviations: MMSE, Mini-Mental State Examination; CI, credible interval.
*Statistically significant with 95% credible interval.

Supplementary Table 4. Effects of genetic markers on the age at the midpoint of cognitive function decline (*M*) and the rate of cognitive function decline (*R*) parameters based on the MMSE after adjusting for sex and education years using the ADNI data (*n* = 385).

Covariates	Minor allele	Regression coefficients on *M*		Regression coefficients on *R*	
		Posterior mean	95% CI	Posterior mean	95% CI
Sex (female vs. male)	—	−1.47	(−3.34, 0.39)	−0.05	(−0.29, 0.18)
Education years	—	−0.04	(−0.36, 0.28)	−0.07	(−0.10, −0.03)*
APOE	ε4	−3.82	(−5.14, −2.44)*	−0.05	(−0.23, 0.13)
ABCA7 rs3764650	G	1.35	(−0.55, 3.22)	0.09	(−0.18, 0.37)
SORL1 rs3737529	T	−1.67	(−5.06, 1.92)	0.16	(−0.44, 1.02)
SORL1 rs1784933	G	1.68	(−1.24, 4.57)	0.11	(−0.30, 0.56)
SORL1 rs2298813	A	2.19	(−0.62, 4.97)	−0.14	(−0.44, 0.18)
BIN1 rs744373	G	−0.13	(−1.50, 1.26)	−0.14	(−0.32, 0.04)
GAB2 rs2373115	A	−0.39	(−1.94, 1.16)	−0.07	(−0.26, 0.14)
CD33 rs3865444	A	0.57	(−0.79, 1.95)	0.12	(−0.06, 0.31)

Note: The sample of 150 AD and 235 MCI progression to AD patients with at least four MMSE measurements were analyzed.
Abbreviations: MMSE, Mini-Mental State Examination; CI, credible interval.
*Statistically significant with 95% credible interval.

Supplementary Table 5. The TaqMan genotyping assay ID for the selected single nucleotide polymorphism (SNP).

Gene	SNP	TaqMan genotyping assay ID
*APOE*_e2/3/4	rs429358	C_3084793_20
*APOE*_e2/3/4	rs7412	C_904973_10
ABCA7	rs3764650	C_27478162_20
SORL1	rs3737529	C_25803133_10
SORL1	rs1784933	C_8799397_10
SORL1	rs2298813	C_16190780_10
BIN1	rs744373	C_1042213_10
GAB2	rs2373115	C_12033202_20
CD33	rs3865444	C_1487395_40
